# First Fully Closed Genome Sequence of Salmonella enterica subsp. enterica Serovar Cubana Associated with a Food-Borne Outbreak

**DOI:** 10.1128/genomeA.01112-14

**Published:** 2014-10-30

**Authors:** Maria Hoffmann, Tim Muruvanda, Cary Pirone, Jonas Korlach, Ruth Timme, Justin Payne, Peter Evans, Jianghong Meng, Eric W. Brown, Marc W. Allard

**Affiliations:** a Office of Regulatory Science, Center for Food Safety and Nutrition, U.S. Food and Drug Administration, College Park, Maryland, USA; b Department of Nutrition & Food Science and Joint Institute for Food Safety & Applied Nutrition, University of Maryland, College Park, Maryland, USA; c Pacific Biosciences, Menlo Park, California, USA

## Abstract

*Salmonella enterica* subsp. *enterica* serovar Cubana (*Salmonella* serovar Cubana) is associated with human and animal disease. Here, we used third-generation, single-molecule, real-time DNA sequencing to determine the first complete genome sequence of *Salmonella* serovar Cubana CFSAN002050, which was isolated from fresh alfalfa sprouts during a multistate outbreak in 2012.

## GENOME ANNOUNCEMENT

*Salmonella enterica* is recognized as one of the most common bacterial agents of food-borne illness causing one million human cases and approximately 400 deaths each year in the United States (1). *Salmonella enterica* subsp. *enterica* serovar Cubana was first identified in Cuba in 1946 (2) and has been associated with several food- and feed-borne outbreaks (3, 4). Presently, only limited information exists regarding the genotypic characterization of *Salmonella* serovar Cubana.

In this report, we announce the availability of a fully closed genome and two plasmids of *Salmonella* serovar Cubana strain CFSAN002050, which was isolated from fresh alfalfa sprouts in Arizona (September 2012). This isolate was obtained by the U.S. Food and Drug Administration as part of a federal public health investigation during a multistate outbreak of *Salmonella* serovar Cubana infections linked to alfalfa sprouts. The illnesses, reported from Arizona, Nevada, and New Mexico, occurred in July and August, 2012 (http://www.fda.gov/Food/RecallsOutbreaksEmergencies/Outbreaks/ucm369067.htm).

Genomic DNA was isolated from overnight cultures using DNeasy blood and tissue kit (Qiagen Inc., Valencia, CA). The pulse-field gel electrophoresis (PFGE) showed that the isolate CFSAN002050 has Xbal pattern JDGX01.0077. The genome was sequenced using the Pacific Biosciences (PacBio) RS II sequencing platform, as previously reported (5). Specifically, a single 10-kb library was prepared according to the PacBio sample preparation methods and sequenced using the C2 chemistry on eight single-molecule real-time (SMRT) cells with a 90-min collection protocol. The 10-kb continuous long read (CLR) data were *de novo* assembled using the PacBio hierarchical genome assembly process (HGAP)/Quiver software package, followed by Minimus2, and polished by Quiver (6). The assembled sequences were annotated using the NCBI Prokaryotic Genomes Automatic Annotation Pipeline (PGAAP) and have been deposited at DDBJ/EMBL/GenBank.

The main chromosome was found to be 4,730,612 bp with a G+C content of 52.2%. In addition, two contigs of 122,863 bp and 166,668 bp representing circular plasmids were also collected. The smaller plasmid encodes genes associated with tellurium resistance and the larger plasmid carries the *tra* genes, which encode functions involved in the conjugative transfer of the plasmid.

Phast analysis (7) identified three intact prophages among *Salmonella* serovar Cubana CFSAN002050. One prophage had 40% overlap with 95% sequence similarity to enterobacterial phage ES18 (NC_006949) and carries the virulence gene *msgA* and phage shock gene *pspG*. Another prophage showed 33% overlap with 99% sequence similarity to prophage Gifsy 1 (NC_101392) and carries the *sopE1* gene that encodes part of the type III secretion system and the virulence gene *msgA* as well. The third prophage had 16% overlap with 96% sequence similarity to *Salmonella* phage vB_SemP_Emek (NC_018275).

The annotated complete genomic sequence is the first of this serovar and provides data from which a better understanding of the evolution and genetics of *Salmonella* serovar Cubana can be gained.

### Nucleotide sequence accession numbers.

The complete genome and plasmids for *Salmonella* serovar Cubana CFSAN002050 are available in GenBank under accession numbers CP006055, CP006056, and CP006057.
